# Independent and Combined Effects of Prenatal Alcohol Exposure and Prenatal Stress on Fetal HPA Axis Development

**DOI:** 10.3390/ijms25052690

**Published:** 2024-02-26

**Authors:** Ludmila N. Bakhireva, Elizabeth Solomon, Melissa H. Roberts, Xingya Ma, Rajani Rai, Alexandria Wiesel, Sandra W. Jacobson, Joanne Weinberg, Erin D. Milligan

**Affiliations:** 1 College of Pharmacy Substance Use Research and Education Center, University of New Mexico Health Sciences Center, Albuquerque, NM 87131, USA; mhroberts@salud.unm.edu (M.H.R.); xinma@salud.unm.edu (X.M.); rajrai@salud.unm.edu (R.R.); ajwiesel@salud.unm.edu (A.W.); 2 Department of Neurosciences, University of New Mexico Health Sciences Center, Albuquerque, NM 87106, USA; bsolomon@salud.unm.edu (E.S.); emilligan@salud.unm.edu (E.D.M.); 3 Department of Psychiatry and Behavioral Neurosciences, Wayne State University School of Medicine, Detroit, MI 48201, USA; sandra.jacobson@wayne.edu; 4 Department of Cellular and Physiological Sciences, Faculty of Medicine, Life Sciences Institute, University of British Columbia, Vancouver, BC V6T 1Z3, Canada; joanne.weinberg@ubc.ca

**Keywords:** prenatal alcohol, stress, pregnancy, placenta, fetal programming, HPA axis

## Abstract

Prenatal alcohol exposure (PAE) and prenatal stress (PS) are highly prevalent conditions known to affect fetal programming of the hypothalamic-pituitary-adrenal (HPA) axis. The objectives of this study were to assess the effect of light PAE, PS, and PAE-PS interaction on fetal HPA axis activity assessed via placental and umbilical cord blood biomarkers. Participants of the ENRICH-2 cohort were recruited during the second trimester and classified into the PAE and unexposed control groups. PS was assessed by the Perceived Stress Scale. Placental tissue was collected promptly after delivery; gene and protein analysis for 11*β*-HSD1, 11*β*-HSD2, and pCRH were conducted by qPCR and ELISA, respectively. Umbilical cord blood was analyzed for cortisone and cortisol. Pearson correlation and multivariable linear regression examined the association of PAE and PS with HPA axis biomarkers. Mean alcohol consumption in the PAE group was ~2 drinks/week. Higher PS was observed in the PAE group (*p* < 0.01). In multivariable modeling, PS was associated with pCRH gene expression (*β* = 0.006, *p* < 0.01), while PAE was associated with 11*β*-HSD2 protein expression (*β* = 0.56, *p* < 0.01). A significant alcohol-by-stress interaction was observed with respect to 11*β*-HSD2 protein expression (*p* < 0.01). Results indicate that PAE and PS may independently and in combination affect fetal programming of the HPA axis.

## 1. Introduction

An estimated 30% of US pregnancies are affected by prenatal alcohol exposure (PAE) [1], and the prevalence of past-month drinking among pregnant persons is 10% according to the national survey estimates [2]. The prevalence of Fetal Alcohol Spectrum Disorders (FASD) among school-age children in the US is reported to be as high as 1–5%, making it one of the most common neurodevelopmental disorders [3]. The 2016 updated clinical guidelines for diagnosing FASD lists self-regulation, which includes impaired stress reactivity and deficits in pain regulation, as one of the key behavioral deficits associated with FASD in infancy [4]; however, human studies in infants are sparse.

In additional to PAE, prenatal stress (PS) has been associated with impaired development and neurodevelopmental and mental health disorders in the child [5,6,7,8,9]. Thus, prenatal psychosocial stress has been conceptualized as a “developmental teratogen” [6,10,11]. From the original research on stress by Hans Selye [12], a working definition of stress as a “state of threatened homeostasis”, and a recognition of stress as a highly heterogeneous construct emerged [13]. Previously proposed conceptual frameworks conceptualized this construct as a stressor itself (acute or chronic), individual perception of stress, and individual response to stress [14]. Of note, prior studies demonstrated that the level of the response may be more related to the *perceived* threat level rather than the actual threat [15], thus assessment of an individual’s perceived burden and unique experience of stressful events has emerged as a common methodological approach to measure psychosocial stress.

The hypothalamic-pituitary-adrenal (HPA) axis is one of the primary systems responsible for the maintenance of homeostasis during stress [16,17]. Prenatal and infancy periods are characterized by increased vulnerability to stressors leading to impaired stress regulation later in life [17]. Preclinical studies indicate that PAE and PS differentially affect glucocorticoid receptors in placentae and fetal brains [18]; however, human studies examining mechanisms leading to specific areas of brain vulnerability are lacking. Both PAE and PS activate the maternal HPA axis and can affect the programming of the fetal HPA axis [18]. Additionally, it is well known that stress plays an important role in alcohol-seeking behavior [19,20]. HPA axis alterations due to PAE persist into adolescence [21], influence executive functioning [22], increase vulnerability for stress-induced changes in neuroimmune function/inflammation, and enhance vulnerability for excessive alcohol consumption in response to stress later in life [23].

During pregnancy, physiological interactions between the mother and fetus are mediated by the placenta, which serves as a key interactive endocrine system linking the maternal and fetal HPA axes [24,25,26,27,28,29,30,31], and both PAE- and PS-induced changes in the placenta alter fetal programming of stress responding. Indeed, a growing literature indicates that both PAE and PS can alter fetal HPA axis activity and regulation, including glucocorticoid regulation of the stress response in the placenta [18,32,33,34,35,36,37,38], leading to lifelong health issues [18,39,40,41]. The amount of cortisol that crosses the placenta is a function of the relative expression of 11-*β* hydroxysteroid dehydrogenases (11*β*-HSD), wherein 11*β*-HSD2 oxidizes active maternal cortisol into inactive 11-dehydrocortisone and 11*β*-HSD1 acts as a reductase and converts inactive 11-dehydro-corticostisone to active cortisol [42]. A key role of placental 11*β*-HSD2 is to reduce levels of maternal cortisol passing to the human fetus [43]. Down-regulation or genetic deficiency of 11*β*-HSD2 is associated with altered glucocorticoid receptor programming [44,45,46]. It is generally accepted that, as gestation progresses, the expression and activity of 11*β*-HSD1 increases and 11*β*-HSD2 decreases to assist with fetal lung maturation; thus, it is important to assess the 11*β*-HSD2/11*β*-HSD1 ratio to account for changes with gestational age [47]. Placenta-derived corticotropin-releasing hormone (pCRH) also plays a crucial role in the programming of the fetal HPA axis [48]. Levels of pCRH increase with gestation [49] and do not follow a circadian rhythm [50]. Unlike hypothalamic CRH, pCRH is stimulated by higher cortisol levels (from maternal and fetal adrenal glands in late gestation), leading to increased maternal and fetal glucocorticoid secretion. The objectives of this study were to focus specifically on measures that would elucidate maternal-fetal HPA activity and interactions, i.e., placental gene and protein expression of two key enzymes that regulate cortisol levels (11*β*-HSD1 and 11*β*-HSD2), placental CRH that stimulates both maternal and fetal HPA activity, and both cortisol and cortisone in umbilical cord blood that provide a readout of how much cortisol is circulating at the time of birth.

(11*β*-HSD2/11*β*-HSD1 ratio and pCRH), and cortisone/cortisol ratio in umbilical cord blood as a downstream measure of fetal HPA axis dysregulation. We hypothesized that both PAE and PS will independently affect the dysregulation of key HPA axis targets in the placenta and result in cortisone/cortisol ratio imbalance in umbilical cord blood. Additionally, we hypothesized that PAE and PS would act synergistically resulting in a multiplicative interaction (effect above and beyond the additive effect of two main effects) with respect to HPA axis targets.

## 2. Results

### 2.1. Sample Characteristics

The mean maternal age at enrollment was 29.5 ± 5.8 years and the mean gestational age at recruitment was 25.2 ± 6.3 weeks (range: 12.0–38.3 weeks). The sample included a large number of minority participants (64.5% Latinx, 6.5% American Indians). Participants across the socio-economic spectrum were included: 35.5% with high school education or less and 41.1% with college degree or higher; 33.9% with family income below $30,000 and 29.0% with income of $70,000 or more. It is of note that there were no differences in maternal socio-demographic characteristics, gestational age at delivery, or infant anthropometric measures between PAE and Control groups (*p*’s > 0.05; Table 1). Significantly higher prenatal stress was observed in the PAE group compared to the Control group at both the second and third trimesters (both *p*’s < 0.01). The PSS-1 and PSS-2 measures were strongly correlated (*r* = 0.75, *p* < 0.001).

As demonstrated in Table 2, in the periconceptional period average alcohol consumption in the PAE group was 0.58 ± 1.11 AAD (equivalent to approximately 8 standard drinks per week), and 83.3% of PAE participants reported at least one binge drinking episode, with 70.8% having ≥2 binge episodes. The mean number of binge episodes during the month around LMP was 3.71 ± 5.75. During pregnancy, the average AAD reported on the TLFB calendars was estimated at 0.001 ± 0.01 (<1 drink/month); however, 89.6% of participants reported at least one binge drinking episode (≥4 drinks/occasion) using a combination of TLFB and targeted questions about episodic binge drinking (e.g., maximum number of drinks consumed in a 24-h period since LMP) [51]. Cumulatively across the periconceptional period and pregnancy, mean AAD in the PAE group was 0.15 ± 0.28 (equivalent to approximately 2 drinks/week). None of the participants had a diagnosis of Alcohol Use Disorder. Mean GGT values, as an indicator of liver function, were similar among PAE and Control groups (14.7 vs. 11.8 U/L, *p* = 0.03); no association between maternal age and GGT was also observed in this population (*p* = 0.23).

There were significant differences in the prevalence of marijuana and tobacco co-exposure between the study groups. Prevalence of marijuana use was 35.4% in the PAE group compared to 10.5% in the Control group (*p* < 0.01). Tobacco use was also much more prevalent in the PAE compared to the Control group (16.7% vs. 1.3%, respectively; *p* < 0.01).

### 2.2. Univariate Analyses

**Figure 1:**
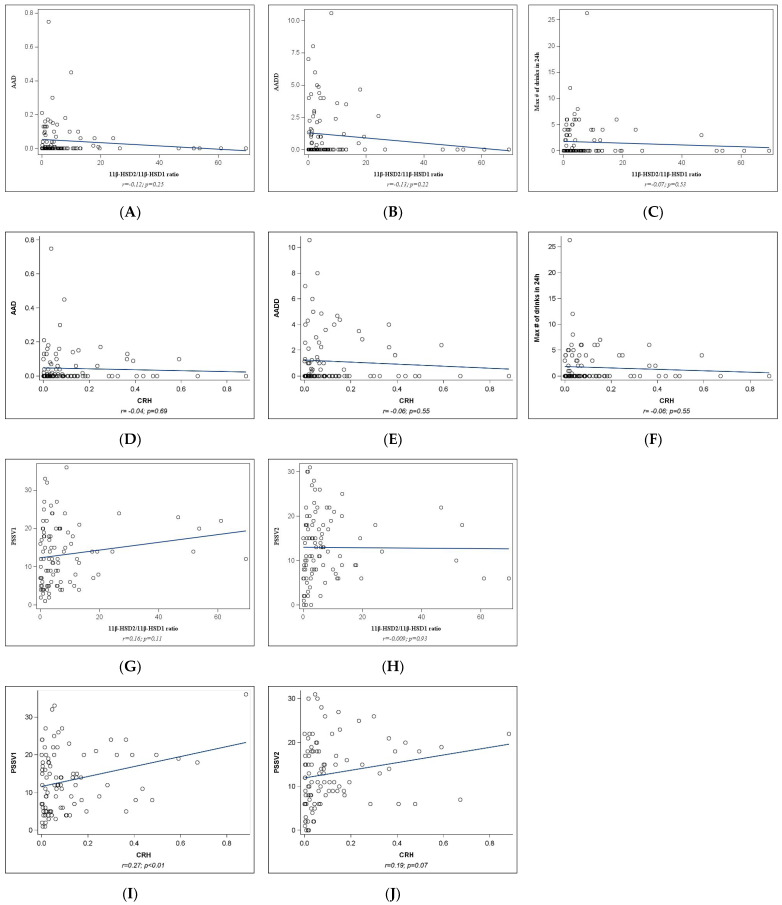
Correlation between prenatal stress, alcohol measures, and placental gene expression of 11*β*HSD-ratio and pCRH. PSS, Perceived Stress Scale administered at Visit 1 (V1) and Visit 2 (V2); AAD, absolute alcohol (oz) per day across pregnancy and periconceptional period; AADD, absolute alcohol (oz) per drinking day across pregnancy and periconceptional period; CRH, placental Corticotropin-releasing hormone; 11*β*-HSD2/11*β*-HSD1 ratio, 11*β*-hydroxysteroid dehydrogenase type 2 over type1 ratio.

**Placental gene analyses**: Correlation analyses between maternal stress (PSS at V1 and V2), alcohol measures (AAD, AADD, maximum number of drinks in a 24 h period), and placental gene expression of the 11*β*HSD-ratio and pCRH are presented in Figure 1. No significant associations with PAE measures or between PSS and 11*β*HSD-ratio were observed (all *p* values ≥ 0.05, Figure 1A–H). Positive correlations were observed between PSS (at both V1 and V2) and pCRH (r = 0.27, *p* < 0.01 and r = 0.19, *p* = 0.07, respectively, Figure 1I,J).

**Figure 2:**
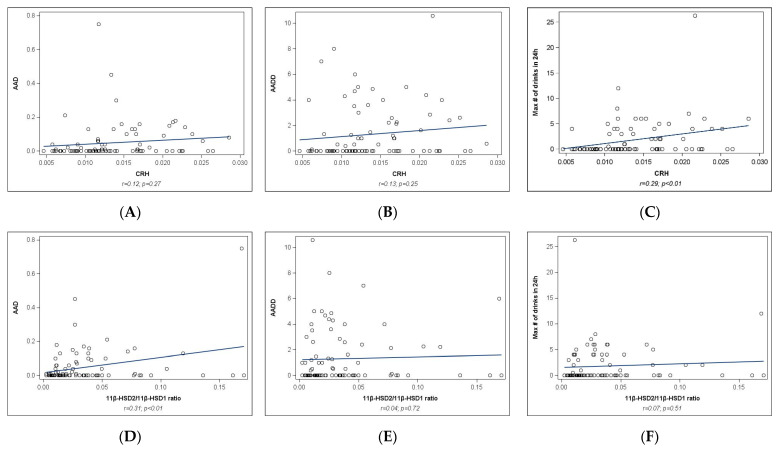

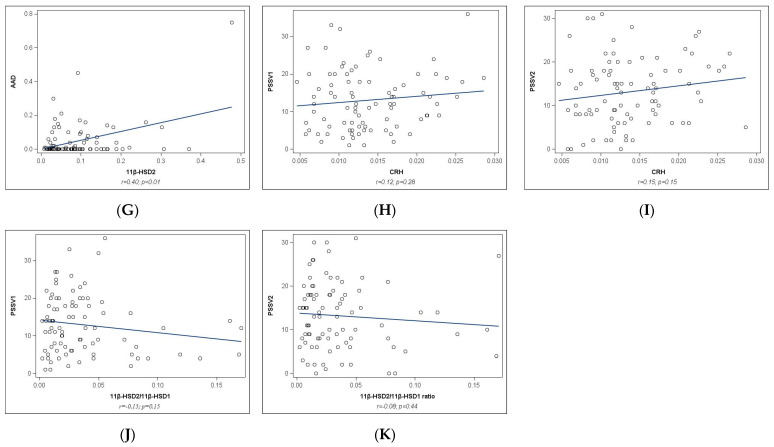
Correlation between prenatal stress, alcohol measures, and placental protein expression of 11*β*HSD-ratio and CRH. PSS, Perceived Stress Scale administered at Visit 1 (V1) and Visit 2 (V2); AAD, absolute alcohol (oz) per day across pregnancy, and periconceptional period; AADD, absolute alcohol (oz) per drinking day across pregnancy and periconceptional period; CRH, placental Corticotropin releasing hormone; HSD11*β*2/HSD11*β*1 ratio, 11*β*-hydroxysteroid dehydrogenase type 2 over type 1 ratio.

**Placental protein analyses**: As shown in Figure 2, no significant associations were observed between mean AAD or AADD and protein expression of pCRH (Figure 2A,B). Significant correlations were observed between maximum number of drinks in a 24 h period and protein expression of pCRH (r = 0.29, *p* < 0.01, Figure 2C), as well as between the mean AAD and protein expression of 11*β*HSD2 (r = 0.40, *p* < 0.01, Figure 2D) and the 11*β*HSD-ratio (r = 0.31, *p* < 0.01, Figure 2G). No significant associations were observed between AADD and 11*β*HSD-ratio (Figure 2E), the maximum number of drinks in a 24 h period, and pCRH (Figure 2F), or with either PSS measure (Figure 2H–K). Table 3 shows that between the PAE and Control group, there were differences in the protein expression of pCRH (*p* < 0.001) and in the protein expression of 11*β*HSD2 (*p* = 0.06).

**Figure 3:**
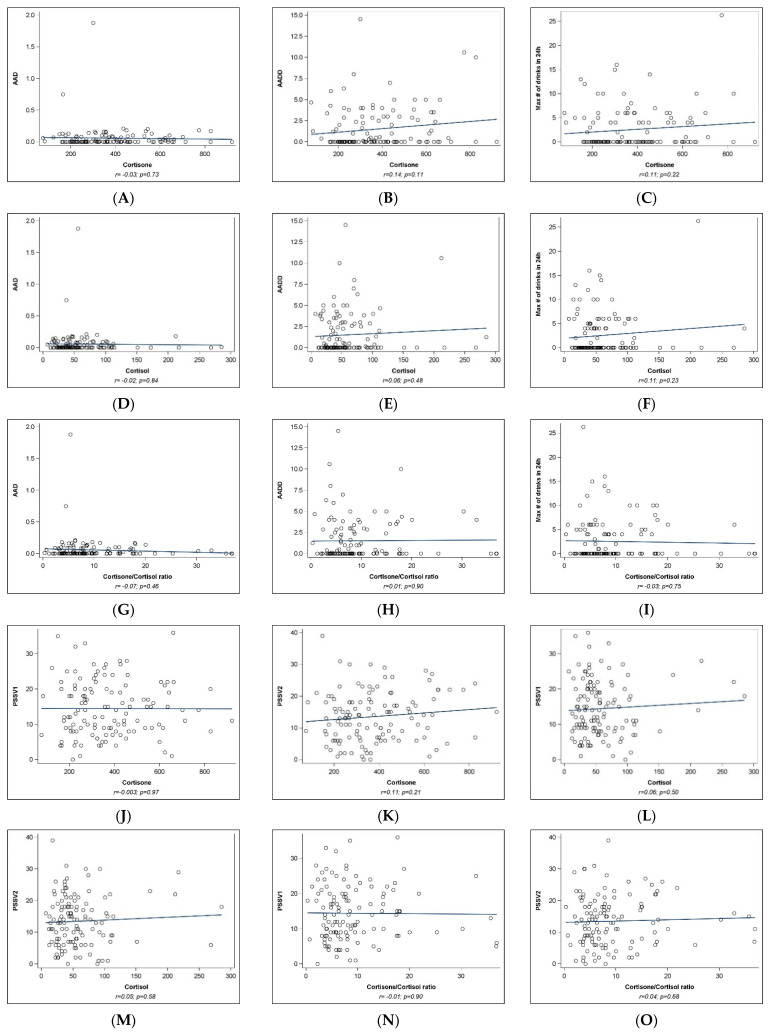
Correlation between prenatal stress, alcohol measures, and umbilical cord blood cortisone, cortisol, and their ratio.

**Umbilical cord analyses**: Correlation analyses are presented in Figure 3. None of the correlation analyses reached statistical significance (Figure 3A–O). A positive association was observed between AADD and cortisone levels in cord blood (r = 0.14); however, the results did not reach statistical significance (*p* = 0.11, Figure 3B).

Table 3 shows that between the PAE and the Control group, there were differences in the protein expression of pCRH (*p* < 0.001) and in the protein expression of 11*β*HSD2 (*p* = 0.058).

### 2.3. Multivariable Analyses

#### 2.3.1. pCRH Expression

As shown in Table 4, PSS-V1 (*β* (SE) = 0.005 (0.002)) was associated with the pCRH *gene* expression in Model 1 (*p* < 0.01). After further adjustment for tobacco and marijuana use (Model 2), and additionally for socio-demographic characteristics (Model 3), PSS-V1 remained significantly associated with pCRH gene expression (*β* (SE) = 0.006 (0.002) and *β* (SE) = 0.006 (0.002), respectively; *p*’s < 0.01).

With respect to pCRH *protein* expression, the maximum number of drinks in a 24 h period (*β* (SE) = 0.0004 (0.0002)) was significantly associated with pCRH protein expression in Model 1 (*p* < 0.01). With further adjustment for tobacco use and marijuana use (Model 2), and additionally for sociodemographic characteristics (Model 3), the association between the maximum number of drinks in a 24 h period and pCRH protein expression remained significant.

#### 2.3.2. Placental 11*β*-HSD2 Expression

Table 5 presents the results of regression analyses for 11*β*-HSD2 protein expression (gene expression results were not included in the multivariable analysis due to a lack of association with either alcohol or PSS measures in univariate analysis). AAD (*β* (SE) = 0.58 (0.12)) and AAD-by-PSS interaction (*β* (SE) = −0.03 (0.01)) were significantly associated with 11*β*-HSD2 protein expression in Model 1 (all *p*’s < 0.01). After further adjustment for tobacco and marijuana use (Model 2), AAD (*β* (SE) = 0.59 (0.11)), AAD-by-PSS interaction (*β* (SE) = −0.02 (0.01)), and tobacco use (*β* (SE) = −0.08 (0.04)) remained significantly associated with 11*β*-HSD2 protein expression. In Model 3, AAD (*β* (SE) = 0.53 (0.11)) and tobacco use (*β* (SE) = −0.09 (0.04)) remained significantly associated with 11*β*-HSD2 protein expression, but the AAD-by-PSS interaction became of borderline statistical significance (*β* (SE) = −0.02 (0.01), *p* = 0.05).

## 3. Discussion

The findings from this study suggest that both maternal stress and prenatal alcohol exposure have an impact on placental physiology, which, in turn, may alter fetal HPA programming. Maternal stress (assessed by PSS at the second and third trimesters) was associated with increased pCRH gene expression, while elevations in corresponding placental protein expression levels were not present. Importantly, however, a robust association was observed between maternal low-level alcohol consumption and increased protein expression of both pCRH and 11*β*-HSD2. This association was observed with categorical measures of PAE (PAE vs. Controls) as well as continuous measures of average alcohol consumption (AAD across pregnancy and periconceptional period) and intensity of drinking (maximum number of drinks in a 24 h period). Results remained significant after adjustment for co-exposures with tobacco and marijuana, and with the exception of placental 11*β*-HSD2 protein expression, after further adjustment for sociodemographic characteristics. There was also a weak but non-significant trend toward a positive correlation between the intensity of drinking (AADD) and cortisone (inactive) in umbilical cord blood.

It is noteworthy that elevated placental 11*β*-HSD2 protein levels were associated with both PAE and prenatal stress, suggesting that the placenta is inappropriately driving the conversion to inactive cortisone in response to the elevated active cortisol induced by stress and alcohol, despite the overall ratio of 11*β*-HSD2/11*β*-HSD1 remaining unchanged. It is possible that the combination of stress and alcohol exerts a greater impact on the converting enzymes, such as 11*β*-HSD2, than either phenomenon alone, resulting in prolonged activation of placental 11*β*-HSD2 at a time when active cortisol is typically increased at parturition. Thus, a lack of association between HPA and stress measures with cortisol and cortisone is observed.

Maternal glucocorticoids can cross the placenta and bind glucocorticoid receptors (GR), which are expressed in the placenta at high levels in decidua, chorion, amnion, and placental villi [52]. Given that glucocorticoids exert profound effects on fetal development via their beneficial actions on cellular functions and the maturation of fetal organ systems, tight regulation of glucocorticoid action occurs to prevent *untimely* excess glucocorticoid exposure in utero [53,54]. As noted previously, 11*β*-HSD1 converts inactive glucocorticoids, such as cortisone, to active glucocorticoids, such as cortisol. Conversely, 11*β*-HSD2 catalyzes active glucocorticoid conversion to inactive cortisone [54]. In the human placenta, 11*β*-HSD2 is predominantly expressed in regions such as fetal membranes and endothelial cells, thus providing appropriately-timed suppression of fetal exposure to the actions of maternally-derived glucocorticoids. In addition, while negative feedback loops involving the GR typically limit maternal plasma glucocorticoids, during pregnancy maternal glucocorticoids stimulate greater placental CRH production, thus the *positive* feedback loop [52]. As pregnancy advances, particularly in the weeks preceding parturition, placental secretion of CRH greatly increases, which drives greater maternal and fetal circulation of glucocorticoids [55,56,57,58]. The data from the present study demonstrate little change in active cortisol, and, in combination with elevated placental 11*β*-HSD2 protein, suggest that the needed and expected elevations in active cortisol are absent. That is, the balance of 11*β*-HSD2 production is dysregulated due to maternal stress and alcohol, and the beneficial maturational role of cortisol on developing fetal systems is thus reduced.

Curiously, data demonstrating elevated 11*β*-HSD2 protein without concurrent elevations in 11*β*-HSD2 gene expression suggest that the source of 11*β*-HSD2 may be extra-placental. Emerging evidence suggests that the placental-glucocorticoid barrier, inclusive of 11*β*-HSD2 activity, may be compromised under pathological conditions or exposure to substances, including alcohol. P-glycoprotein (P-gp) is a highly abundant efflux transporter of several factors of toxicants and xenobiotics, and is expressed on placental trophoblast cells; it has been characterized to pump glucocorticoids back into maternal circulation. Reduced P-gp expression opens the placental-glucocorticoid barrier, and a reduction in placental P-gp increases fetal exposure to glucocorticoids [59]. A recent review that discussed the actions of P-gp on the blood-placenta barrier suggests that pathological conditions during pregnancy during which the blood-placental barrier is compromised, such as preeclampsia, or following proinflammatory exposures, expression of P-gp is significantly decreased [60]. These findings together with the current data suggest that under maternal stress and alcohol exposure, the blood-placental barrier may be compromised contributing to inappropriate placental and fetal glucocorticoid regulation and exposure known to profoundly influence fetal HPA programing.

Clinical and preclinical studies of PAE demonstrated that alcohol interacts with the activation of the HPA axis [18,61,62,63]. Even moderate levels of alcohol consumption increase plasma glucocorticoids because alcohol causes increased CRH release from the hypothalamus, likely by direct interaction of ethanol with the CRH gene promoter [64,65]. Additionally, elevated basal and stress-induced fetal glucocorticoids have been observed, even in response to relatively low alcohol levels of consumption [66]. Reports of secondary outcomes from PAE in preclinical models that test stressor sensitivity show corresponding elevations in plasma glucocorticoids in parallel with anxiety-like behavior after exposure to chronic mild stress [67]. Clinically, evidence supports that adverse childhood experiences increase the prevalence of adverse HPA activation to a greater extent in children with FASD [68]. Combined, published evidence supports that the actions of maternal glucocorticoids from increased HPA axis activation as a consequence of maternal stress and/or alcohol exposure directly impact the placenta with resultant amplification of fetal HPA axis activation [56,69]. A recent clinical report demonstrated that PAE was associated with a greater prevalence of mental health disorders during middle adulthood, and noted that efforts aimed at mitigating these outcomes should be directed at the early identification of PAE and reducing environmental stressors later in life [70]. Additionally, placental epigenetic mechanisms are known to affect HPA axis signaling. For example, hypermethylation of the NR3C1 gene encoding glucocorticoid receptors has been associated with both prenatal stress and PAE (reviewed by Ruffaner-Hanson et al., 2022) [52]. Additionally, epigenetic mechanisms in the placenta (DNA methylation, miRNA expression) have been linked to future neurodevelopmental outcomes (reviewed by Lester and Marsit, 2018) [71].

It is important to emphasize that the observed alterations in placenta biomarkers were observed at very low levels of alcohol exposure. The ENRICH-2 cohort focused on light-moderate PAE by design given the limited and conflicting evidence with respect to the effects of exposure to such lower levels in prior research. While in the periconceptional period (1 month around LMP), participants reported consumption of approximately 8 standard drinks per week and over 70% reported having at least 2 binge episodes, the average consumption during pregnancy dropped substantially. In fact, average estimates from the TLBF calendars were less than 1 drink per month, which has traditionally been considered negligible. However, we have previously demonstrated that pregnant persons might disclose alcohol use during pregnancy more openly if they are asked targeted questions, such as the maximum number of drinks consumed in a 24 h period since LMP and the number of drinks last time they consumed alcohol (with the date of last drink being compared to LMP) [51]. In fact, using such targeted questions and data from TLFB interviews, almost 90% of participants in the PAE group reported at least one binge drinking episode since LMP. Thus, the level of consumption in this cohort represented mild-moderate use in the periconceptional period and low average consumption with episodic binge drinking during pregnancy. Cumulatively across the periconceptional period and pregnancy, participants in the PAE group consumed approximately 2 drinks/week. We are not aware of any prior studies that demonstrated an association between that level of PAE and placenta measures of HPA axis function (robust association with pCRH and 11*β*-HSD2 protein expression in this study). These novel findings have important clinical implications demonstrating that even low levels of PAE might affect fetal programming of key neurobiological systems predisposing children to adverse neurobehavioral outcomes. We have demonstrated earlier that neurobehavioral deficits associated with lower levels of PAE might not be apparent early in life [72]; however, deficits in the infant’s autonomic system dysregulation [73] and, as demonstrated by the present study, alterations in placental biomarkers might be identified shortly after birth.

The results of this study, however, should be interpreted in light of its limitations. First, we acknowledge that the sample size was relatively small, limiting our ability to detect smaller effect sizes and examine more complex interactions beyond PAE and prenatal stress. The limited sample size also did not allow for stratification of results by infant sex. Second, while the effects of prenatal tobacco and marijuana use (common co-exposures with alcohol) and socio-demographic factors have been carefully accounted for in multivariable analyses, we acknowledge that there could be additional risk and resilience factors that affect the HPA axis. Third, as described in the Introduction, “prenatal stress” is a complex multi-dimensional construct. In this analysis, we chose to focus on perceived stress, assessed by a widely validated scale administered repeatedly during the second and third trimester of pregnancy, as a measure of individual perception or individual response to stress. We acknowledge that other constructs of stress (e.g., assessment of chronic and acute “stressors”, maternal adverse childhood experience, pregnancy-specific stress), physiological and maladaptive responses (e.g., maternal psychopathology), and an array or additional risk and mitigating factors (e.g., psycho-social support, coping strategies, physical activity, genetic vulnerability, general medical and reproductive health) might affect fetal programming of the HPA axis [14,74,75,76,77,78]. Additionally, given that PSS captured perceived stress levels in the past 30 days, and participants were recruited during the second trimester, perceived stress during the first trimester was not captured. However, the close correlation between PSS scores at study visits 1 and 2 (r = 0.75, *p* < 0.001) provides some reassurance that the construct was relatively stable over the course of pregnancy with minimal within-subject variability. Future studies should further examine the effect of different “prenatal stress phenotypes” on measures of maternal-fetal HPA activity [79].

Fourth, placental analyses were limited to sections of chorionic villus tissue.

While placental villi are composed of trophoblast lineage cells and share genetic material mostly with the fetus, thus appropriate for investigation of fetal programming of the HPA axis, a comparison of all three layers of the placenta (basal plate—mostly maternal composition, chorionic villus, and chorionic plate—mostly fetal composition) would offer a more comprehensive interpretation of the results and understanding of maternal-fetal interface. Future studies are needed to identify the specific cell types across all layers of the placenta that are driving the observed gene expression signature. Finally, while this study focused on key measures of maternal-fetal HPA activity and interactions (placental gene and protein expression of 11*β*-HSD1, 11*β*-HSD2, and pCRH, cortisol, and cortisone in umbilical cord blood), subsequent studies can build on our data and extend the investigation to other important markers, such as the glucocorticoid receptors and epigenetic mechanisms.

Strengths of the present study include a prospective study design with a rigorous assessment of PAE by repeated TLFB interviews, targeted questions regarding binge drinking behavior, and a comprehensive battery of ethanol biomarkers assessed in the pregnant person and infant. Also, it is important to note that the effect of PAE on the HPA axis was examined in parallel with the effect of maternal perceived stress—a known major factor affecting fetal programming of the HPA axis, as well as their interactive effects. Another strength of the study is a comprehensive assessment of placental gene and protein expression in samples collected shortly after birth, as well as downstream measures of HPA axis function in umbilical cord blood.

In summary, the novel findings from this study suggest that PAE and prenatal stress may independently and in combination affect fetal programming of the HPA axis. These data are the first investigation of the effects of PAE and PS on HPA function in a human cohort and contribute novel and important information to the field. The HPA axis (particularly, the corticotropin-releasing hormone receptor) plays a unique role in mediating endocrine and behavioral responses to stress and regulating alcohol consumption [23]. HPA axis alterations due to PAE persist into adolescence [21], influence executive functioning [22], and enhance vulnerability to excessive alcohol consumption in response to stress later in life [23]. Our focus on the placenta, as a key interactive endocrine entity linking the maternal and fetal HPA axes [24,25,26,27,28,29,30,31], lays the foundation for better understanding the mechanisms underpinning neurodevelopmental outcomes associated with PAE and prenatal stress. Placenta and cord blood biomarkers are also important diagnostic modalities offering an opportunity to identify children at risk of adverse outcomes [80].

Heterogeneity in specific approaches under each non-pharmacologic modality and variability in participants’ fidelity to a specific intervention makes it difficult, however, to make unequivocal conclusions about effectiveness. In addition to mindfulness-based stress reduction approaches, pharmacologic treatment might be needed for pregnant patients experiencing symptoms of depression, anxiety, or other impaired mental health disorders. The American College of Obstetricians and Gynecologists (ACOG) Clinical Practice Guidelines provide recommendations on the management of perinatal mental health conditions with a focus on psychopharmacotherapy [81]. With respect to preventive efforts to reduce PAE, screening and brief intervention are widely recommended approaches [82]. Additionally, motivational interview-based approaches as preconception services in primary care settings, such as Project CHOICES, have demonstrated effectiveness in reducing the risk of alcohol-affected pregnancies [83,84]. Future studies need to focus on examining the effectiveness of novel intervention approaches to stress reduction and abstinence from alcohol during pregnancy.

The evidence of elevated 11*β*-HSD2 from PAE and prenatal stress presents a potential novel therapeutic target, whereby adverse effects of exposure might be attenuated by inhibiting 11*β*-HSD2 either directly or indirectly via upstream pathways or epigenetic modifications. Future targets for analysis might include proteins confirmed to have a positive [p38 Mitogen-activated protein kinases (MAPK)], or negative [extracellular signal-regulated kinase (ERK)1/2 MAPK], effect on the regulation of placental 11*β*-HSD2 [85]. Medications known to alter DNA methylation or histone modifications as well as non-coding RNAs (e.g., miR-24-3p) may also provide indirect changes in the regulation of 11*β*-HSD2 [86,87], highlighting another area for future studies. Future studies should also link placental biomarkers to higher-order deficits and secondary disabilities in children with PAE (e.g., executive functioning, impulsivity, metabolic disorder, obesity) and examine the sensitivity and specificity of placenta and cord blood biomarkers with respect to these long-term outcomes.

## 4. Methods and Materials

### 4.1. Study Design and Population

The ENRICH-2 pre-birth cohort was conducted at the University of New Mexico (PI: Bakhireva). It employed a prospective design, which included four study visits (V): (V1) baseline prenatal visit during the second trimester (12–27.9 gestational weeks); (V2) early third-trimester visit (28–32 weeks); (V3) collection of maternal (blood, urine), infant (blood spots), delivery specimens (placenta, umbilical cord blood) and infant-caregiver assessment hospital stay for labor and delivery; and (V4) infant-caregiver assessment at 6 months after birth. Participants were recruited into the two study groups: PAE and healthy controls (HC). Eligibility criteria for all study groups were as follows: (1) at least 18 years of age; (2) singleton pregnancy; (3) gestational age at recruitment: 12–38 weeks, (4) planning to deliver at UNM Hospital (UNMH), (5) planning to reside in New Mexico for 6 months after delivery; and (6) ability to provide written consent in English; (7) no prenatal use of cocaine, methamphetamines, opioids/medication-assisted therapy or MDMA (per self-report, positive urine drug tests, or medical records review). Additionally, after delivery, additional eligibility criteria were applied: (a) gestational age at delivery ≥35 weeks; (b) no fetal/neonatal diagnosis of a major structural abnormality or severe complication in the newborn period; (c) no maternal or newborn administration of corticosteroids. All participants gave written informed consent.

In the ENRICH-2 cohort, 168 study participants who met all eligibility criteria completed V3. Of those, placental samples were collected from 144 individuals (86% collection rate) and cord blood from 154 individuals (92% collection rate). For the placental analysis, samples with collection/processing time > 6 h from delivery (*n* = 46) and samples from a participant who withdrew from V3 (*n* = 1) or had severe neonatal complications (*n* = 1) were excluded, resulting in 96 samples transferred for analyses. After excluding outliers (1 for gene analysis and 2 for protein) and missing data (7 for protein), the final placenta sample size was 95 for gene analysis and 87 for protein analysis. For the umbilical cord blood analyses, samples from participants not meeting continuous eligibility criteria (*n* = 24) or receiving corticosteroids before labor (*n* = 1) were excluded, as were samples from participants with infants with severe neonatal complications/birth defects (*n* = 3). After excluding 2 outliers at the analysis stage, the final sample size for cord blood biomarkers was 124.

### 4.2. Assessment of PAE and PS

PAE was ascertained by four repeated Timeline Follow-Back (TLFB) interviews—the “gold standard” in the field [88,89], and a comprehensive panel of ethanol biomarkers. Alcohol consumption was assessed at V1–V3 and captured alcohol use in the periconceptional period (TLFB_1_), second trimester (TLFB_2_), early third trimester (TLFB_3_), and 30 days before delivery (TLFB_4_). For each calendar and across pregnancy, the following summary measures were estimated: absolute ounces of alcohol (AA) per day, AA/drinking day, % of drinking days, and number of binge episodes. One AA corresponds approximately to 0.5 standard drink units (SDU).

At V1 and V3 maternal blood and urine samples were collected for analysis of maternal biomarkers. Maternal samples were analyzed at both visits for γ-glutamyltranspeptidase (GGT), carbohydrate-deficient transferrin (%dCDT), phosphatidylethanol (Peth), and urine ethyl sulfate/ethyl glucuronide (uEtS/uEtG). Additionally, during the routine newborn heel lancing, two blood spots were collected for analysis of Peth in dry blood spots (Peth-DBS) at V3. These biomarkers have different detection windows (i.e., 1–2 months for GGT; 4–5 weeks for %CDT, 2–4 weeks for Peth, and <4 days for uEtG/uEtS); thus, they can capture different patterns of alcohol use and improve sensitivity and specificity of PAE identification [90].

To be classified into the PAE group, participants had to meet the following criteria: (1) AUDIT-C score ≥ 2 or report ≥ 2 binge episodes during the periconceptional period (1 month around last menstrual period [LMP]; (2) more than minimal-risk alcohol use (>13 SDU/month); (3) at least one positive ethanol biomarker. To be classified into the HC group, participants had to meet the following criteria: (1) score < 2 on the AUDIT-C, no binge episodes, and ≤13 SDU in the periconceptional period; (2) no alcohol use in pregnancy (TLFB_2–4_); (3) negativity on all biomarkers. These criteria are consistent with the definition of PAE in the DSM-5 guidelines for Neurodevelopmental Disorder associated with PAE (ND-PAE) [88]. It is important to note that the innovative aspect of the ENRICH cohort was our focus on mild-to-moderate PAE. None of the participants were diagnosed with Alcohol Use Disorder.

Maternal psychosocial PS was assessed by the 10-item Perceived Stress Scale (PSS) [91] administered at V1 (second trimester) and V2 (third trimester). The PSS assessed perceived stress during the past 30 days before each visit as a measure of current stress. PSS is a widely-used global measure of stress that assesses the degree to which life is perceived as unpredictable, uncontrollable, and overloading, as well as how well an individual feels they can cope with external demands [91,92]. The higher score indicates higher perceived stress and lower coping abilities. The PSS has been in use since 1982 and remains the most validated and widely used tool to assess the perception of stress. A recent systematic review and meta-analysis of 76 different samples (total sample size for PSS-10 was 46,053) demonstrated highly satisfactory psychometric results of the PSS with very high model-data fit [93].

### 4.3. Socio-Demographic Characteristics, Prenatal Environment, and Other Substances

Maternal demographic characteristics, medical and reproductive history, and socio-economic environment were ascertained at V1. At V1–V3, any use of nicotine products (cigarettes, e-cigarettes, nicotine replacement therapy) cannabis, cocaine, opioids, methamphetamine, MDMA, inhalants, benzodiazepines, and sedatives was ascertained by trained interviewers. Street names of drugs were provided to facilitate recall. Additionally, maternal urine collected at V1 and V3 was sent to the US Drug Testing Laboratory for analysis of a Drug Panel 7 (amphetamines, barbiturates, benzodiazepines, cannabinoids, cocaine, opiates, and propoxyphene). Participants who tested positive for opioids, methamphetamines, MDMA, or cocaine were disenrolled, while nicotine and cannabis were accounted for in multivariable analyses.

### 4.4. Placental Tissue and Cord Blood Collection

After enrollment, communication orders were placed in participants’ electronic medical records, so participants could be flagged during the admission for labor and delivery. A research team closely monitored obstetrics admissions, including during weekends and observed holidays. Placenta samples were collected immediately after delivery or refrigerated at +4 °C for up to 6 h before processing by the research team (mean collection time was 3.0 ± 1.4 h). After removing the decidua basalis and amniochorionic membrane, sections of villus tissue (spongy tissue located directly under the basal plate, which shares genetic material mostly with the fetus) were dissected avoiding major vessels and areas with calcifications or other abnormalities. A mechanism was in place to obtain research-specific sections prior to routing placental samples for pathology examination, when clinically indicated, thus minimizing potential sampling bias. Dissection tools were wiped with RNaseZAP solution. Sections of placenta tissue were rinsed in saline, blotted to remove excess blood and fluid, placed in polypropylene tubes containing RNAlater stabilizing solution, flash frozen with liquid nitrogen, and stored in air-tight containers at −80 °C until analysis. Umbilical cord blood (4 mL in EDTA tube) was collected after delivery; plasma was separated by centrifugation within 3 h of collection, and at −80 °C until analysis.

### 4.5. mRNA and Protein Initial Homogenization

Frozen placental sections were cut into smaller fragments using a scalpel and forceps on a glass dish over ice. Tissue fragments weighing between 20–80 mg were then homogenized in a proportional volume (100 μL per 10 mg tissue) of mRNA Lysis Buffer (AM1560, ThermoFisher, Waltham, MA, USA). RNA homogenates were flash-frozen in liquid N_2_ and stored at −80 °C. 50–100 mg of remaining tissue, now beginning to thaw, was homogenized in 200 μL of ice cold homogenization buffer (HB) (20 mM Tris, 1 mM EDTA, 320 mM Sucrose, 0.2 mM Sodium Orthovanadate, and 1:100 Halt™ Protease Inhibitor Cocktail (78429, ThermoFisher, Waltham, MA, USA), 1M Hexylene Glycol, 20 μM Digitonin, pH adjusted to 7.4 with 1N HCl) [29,94,95,96,97,98]. All homogenizations were carried out with disposable tissue grinders and an electric pestle rotator, on ice.

### 4.6. Cytosolic and Nuclear Protein Fractionation

Immediately following homogenization, placental homogenates were spun at 1300× *g* at +4 °C for 6 min; supernatant removed and saved. The pellet was resuspended in volume proportional to tissue weight (50 μL per 10 mg) of HB. The resuspended pellet was spun at 1300× *g* at +4 °C for 10 min; supernatant combined with previous supernatant, yielding cytosolic protein enriched lysate. Cytosolic protein lysates and pellets were flash-frozen and stored at −80 °C. Pellets were resuspended with ice cold Nuclear Extraction Buffer (NEB) (20 mM Tris, 1 mM EDTA, 320 mM Sucrose, 0.2 mM Sodium Orthovanadate, and 1:100 Halt™ Protease Inhibitor Cocktail (78429, ThermoFisher, Waltham, MA, USA), 75 mM KCl, 75 mM NaCl, 1% (*v*/*v*) NP-40, 0.5% (*w*/*v*) Sodium Deoxycholate, 0.1% SDS, 5 mM DTT, pH adjusted to 7.4 with 1N HCL) [99]. NEB volume was proportional to tissue weight (5 μL per 10 mg tissue). To resuspended pellets, 100 μ/mL of Collagenase type 2 was added, then incubated at 37 °C for 30 min [100]. The digested pellet was then transferred to a Bioruptor tube and sonicated in a BioruptorPlus (Diagenode, Denville, NJ, USA) machine for 20 cycles of 30 s on and 90 s off at high power at +4 °C. The sonicated sample was then spun at 15,000× *g* at +4 °C for 10 min. The supernatant, enriched with nuclear proteins, was removed, flash-frozen, and stored at −80 °C. Protein concentrations were quantified by Bradford assay (#23200, ThermoFisher, Waltham, MA, USA).

### 4.7. Gene Expression qPCR Analysis

RNA homogenates were processed with the *mirV*ana Total RNA Isolation kit following the manufacturer’s protocol and stored at −80 °C. 500 ng of RNA was converted to cDNA with QuantiTect Reverse Transcription Kit (205311, Qiagen, Hilden, Germany), and total RNA and cDNA were quantified with a Qubit 2.0 Fluorometer. cDNA dilution was optimized for each primer pair. qPCR standards and primers manufactured by GenScript and Integrated DNA Technologies (IDT, Coralville, IA, USA). All qPCR assays were run on LightCycler96 Machine (Roche, Basel, Switzerland) with FastStart essential green master mix (Roche, Basel, Switzerland). All primers were validated by melt curve and gel electrophoresis. Refer to Supplemental Table S1 for primer and standard oligo details. A standard curve was run on each 96-well plate [101,102,103]. Eleven housekeeping genes were evaluated by qPCR for stability, of which Glyceraldehyde-3-phosphate dehydrogenase (GAPDH), Beta-2-microglobulin (B2M), and Actin-related protein 2/3 complex subunit 3 (ARPC3) were revealed to be the most stable [104,105]. A geometric mean of the 3 housekeeping genes expression was used to normalize target gene data [106].

### 4.8. Placenta Protein Analysis and Umbilical Cord Hormones

Cytosolic fraction of target proteins (11b-HSD Type 1, 11b-HSD type 2, and pCRH) was measured with ELISA kits from LifeSpanBio (LS-F32857, LS-F8771, LS-F39197, Bhopal, India). Assays were optimized and run following the manufacturer’s protocol. Plasma derived from umbilical cord blood was analyzed for cortisol and cortisone with ELISA assay kits from Arbor Assays (K003-H1, K017-H1, Ann Arbor, MI, USA). Assays were optimized and run following the manufacturer’s protocol.

### 4.9. Statistical Analysis

Maternal sociodemographic and clinical characteristics for the study population are described using means (±standard deviation [SD]) for continuous measures and frequencies (percentage) for categorical measures. Differences between the study groups (PAE, HC) for sociodemographic and medical characteristics, prenatal stress, substance use, and HPA axis measures (11*β*-HSD2/11*β*-HSD1 ratio, pCRH, cortisone/cortisol ratio) were compared using chi-square or Fisher’s exact test for categorical variables and *t*-test or Kruskal–Wallis test for continuous variables, as appropriate. Pearson correlation analyses were used to examine the association between PSS assessed at V1 and at V2, as well as correlations between placenta measures and maternal stress (PSS at V1 and V2), alcohol measures (AAD, AADD, maximum number of drinks in a 24 h period).

For alcohol exposure measures that correlated with HPA axis biomarkers at least at the *p* < 0.10 level, multivariable linear regression models were conducted. In the initial multivariable model (Model 1), only alcohol exposure measure, prenatal stress, and their interaction were included. Subsequently, regression models were further adjusted for prenatal tobacco and marijuana use (Model 2), and then additionally adjusted for sociodemographic factors—maternal race, ethnicity, and education level (Model 3). A specific alcohol exposure measure included in regression models was determined based on results from correlation analyses. Akaike information criterion (AIC) was used to decide on the inclusion of an interaction term beyond Model 1 (the lowest AIC model presented). Sensitivity analyses were conducted to exclude study participants who later were disqualified based on PAE/HC criteria (see Supplemental Table S2). Statistical tests were two-sided, with a *p*-value less <0.05 considered statistically significant. All statistical analyses were conducted using SAS version 9.4 (SAS Institute, Cary, NC, USA).

## Figures and Tables

**Table 1:** Demographic characteristics by study group (*n*= 124).

	Control (*n* = 76)	PAE (*n* = 48)	*p*
Maternal characteristics			
	Mean ± SD	Mean ± SD	
Maternal age at enrollment (years)	29.7 ± 5.7	29.2 ± 5.9	0.63 ^1^
Years of education (years)	14.7 ± 3.5	14.8 ± 3.5	0.97 ^1^
	N (%)	N (%)	
Marital status:			
Single/separated/divorced	15 (19.7)	15 (31.3)	0.14 ^2^
Married/cohabitating	61 (80.3)	33 (68.8)	
Ethnicity (Hispanic/Latinx)	47 (61.8)	33 (68.8)	0.43 ^2^
Race:			
White	51 (67.1)	37 (77.1)	0.82 ^3^
Black or African American	3 (4.0)	1 (2.1)	
American Indian or Alaskan Native	5 (6.6)	3 (6.3)	
Multi-racial/other	13 (17.1)	5 (10.4)	
Prefer not to report	4 (5.3)	2 (4.2)	
Education:			
High school or less	28 (36.8)	16 (33.3)	0.48 ^2^
Some college or vocational school	15 (19.7)	14 (29.2)	
College degree or higher	33 (43.4)	18 (37.5)	
Family income *:			
Under $30,000	25 (32.9)	17 (35.4)	0.57 ^2^
$30,000–49,000	13 (17.1)	13 (27.1)	
$50,000–69,000	13 (17.1)	5 (10.4)	
$70,000 or over	24 (31.6)	12 (25.0)	
Currently employed	46 (60.5)	30 (62.5)	0.83 ^2^
Health insurance:			0.90 ^2^
Employer-based insurance	30 (39.5)	18 (37.5)	
Medicaid	27 (35.5)	19 (39.6)	
Other **	19 (25.0)	11 (22.9)	
Maternal prenatal stress:			
PSS-V1 (2nd trimester)	12.8 ± 7.8	17.0 ± 7.2	<0.01 ^1^
PSS-V2 (3rd trimester)	11.8 ± 7.8	16.2 ±7.2	<0.01 ^1^
Perinatal and infant outcomes			
	Mean + SD	Mean + SD	
Gestational age at birth (weeks)	39.0 ± 1.3	39.1 ± 1.3	0.58 ^1^
Birth weight percentile	48.1 ± 28.8	50.8 ± 27.2	0.60 ^1^
Birth height percentile	61.4 ± 28.7	63.9 ± 29.5	0.53 ^1^
OFC percentile	55.5 ± 29.1	57.3 ± 29.4	0.81 ^1^

PSS, Perceived Stress Scale administered at visit 1 (V1) or visit 2 (V2); OFC, occipital frontal circumference. * 2 subjects have missing data. ** No insurance, self-purchased, or other public insurance. ^1^ Based on Mann–Whitney test. ^2^ Based on chi-square test. ^3^ Based on Fisher’s exact test.

**Table 2:** Alcohol and substance use by study group (*n*= 124).

	Control (*n* = 76)	PAE (*n* = 48)
Periconceptional period		
AAD, Mean ± SD	0.01 ± 0.02	0.58 ± 1.11
AADD, Mean ± SD	0.09 ± 0.27	2.19 ± 1.38
Maximum number of drinks in 24 h, Mean ± SD	0.07 ± 0.30	6.46 ± 4.73
Any binge drinking episodes, Mean ± SD	0.00 ± 0.00	3.71 ± 5.75
Any binge drinking episodes, *n* (%)	0 (0.0)	40 (83.3)
≥2 binge episodes, *n* (%)	0 (0.0)	34 (70.8)
≥2 positive biomarkers, *n* (%)	0 (0.0)	2 (4.2)
During pregnancy		
AAD, Mean ± SD	0.00 ± 0.00	0.001 ± 0.01
AADD, Mean ± SD	0.00 ± 0.00	0.03 ± 0.10
Maximum number of drinks in 24 h, Mean ± SD	0.00 ± 0.00	0.16 ± 0.41
Any binge drinking episodes, *n* (%)	0 (0.0)	43 (89.6)
≥2 positive biomarkers, *n* (%)	0 (0.0)	1 (2.1)
Across pregnancy and periconceptional period		
Mean AAD, Mean ± SD	0.00 ± 0.01	0.15 ± 0.28
Mean AADD, Mean ± SD	0.17 ± 0.53	3.66 ± 2.83
Maximum number of drinks in 24 h, Mean ± SD	0.07 ± 0.30	6.46 ± 4.73
Any binge drinking episodes, *n* (%)	0 (0.0)	44 (91.7)
≥2 binge episodes, *n* (%)	0 (0.0)	34 (70.8)
≥2 positive biomarkers, *n* (%)	0 (0.0)	7 (14.6)
Other substances during pregnancy		
Marijuana, *n* (%)	8 (10.5)	17 (35.4)
Tobacco, *n* (%)	1 (1.3)	8 (16.7)

AAD, absolute alcohol (ounces) per day [1 AA is equivalent to ~0.5 standard drinks]; AADD, absolute alcohol per drinking day.

**Table 3:** Placenta CRH and 11*β*-HSD2 expression and HPA axis measures in umbilical cord blood by study group.

	Control	PAE	*p* ^1^
	Mean ± SD	Mean ± SD	
Placenta (*n* = 95 for gene expression, *n* = 87 for protein expression) *			
Gene pCRH	0.11 ± 0.17	0.12 ± 0.15	0.62
Protein pCRH	0.01 ± 0.01	0.02 ± 0.01	<0.001
Gene 11*β*-HSD2	0.07 ± 0.09	0.06 ± 0.07	0.84
Protein 11*β*-HSD2 *	0.08 ± 0.07	0.11 ± 0.10	0.058
Umbilical cord blood (*n* = 124)			
	(*n* = 76)	(*n* = 48)	
Cortisone	366.9 ± 161.9	384.7 ± 183.3	0.62
Cortisol	58.4 ± 44.8	59.0 ± 47.5	0.70
Cortisone/Cortisol ratio	9.2 ± 7.4	9.5 ±6.9	0.67

* N for gene expression: 66 Controls, 29 PAE; N for protein expression: 58 Controls, 29 PAE. ^1^ Based on Mann–Whitney test.

**Table 4:** Predictors of pCRH expression: results of multivariable linear regression.

	PSS-V1	AAD	Tobacco	Marijuana
	*β* (SE)	*β* (SE)	*β* (SE)	*β* (SE)
Gene pCRH (*n* = 95)				
Model 1	0.005 (0.002) **	−0.05 (0.15)	--	--
Model 2	0.006 (0.002) **	0.01 (0.16)	−0.05 (0.08)	−0.04 (0.04)
Model 3	0.006 (0.002) **	0.004 (0.17)	−0.04 (0.09)	−0.03 (0.04)
Protein pCRH (*n* = 87)				
	PSS-V1	Max number of drinks in 24 h	Tobacco	Marijuana
	*β* (SE)	*β* (SE)	*β* (SE)	*β* (SE)
Model 1	0.0001 (0.0001)	0.0004 (0.0002) **	--	--
Model 2	0.0001 (0.0001)	0.0003 (0.0002) *	−0.002 (0.003)	0.002 (0.002)
Model 3	0.0001 (0.0001)	0.0004 (0.0002) *	−0.001 (0.003)	0.002 (0.002)

AAD, absolute alcohol (ounces) per day; PSS-V1, Perceived Stress Scale at visit 1. * *p* < 0.05, ** *p* < 0.01. Model 1: Alcohol measure and PSS-V1. Model 2: Alcohol measure, PSS-V1, tobacco use, marijuana use. Model 3: Alcohol measure, PSS-V1, tobacco use, marijuana use, race, education, and Hispanic ethnicity.

**Table 5:** Predictors of placental 11*β*-HSD2 protein expression: results of multivariable linear regression.

	PSS-V1	AAD	PSSV1 *AAD	Tobacco	Marijuana
	*β* (SE)	*β* (SE)	*β* (SE)	*β* (SE)	*β* (SE)
		Protein 11*β*-HSD2 (*N* = 87)			
Model 1	0.0002(0.001)	0.58(0.12) **	−0.03(0.01) **	--	--
Model 2	0.0003(0.001)	0.59(0.11) **	−0.02(0.01) *	−0.08(0.04) *	−0.02(0.02)
Model 3	0.0004(0.001)	0.53(0.11) **	−0.02(0.01)	−0.08(0.04) *	−0.02(0.02)

AAD, absolute alcohol (oz) per day; PSSV1, Perceived Stress Scale at visit 1. * *p* < 0.05, ** *p* < 0.01. Model 1: Alcohol measure AAD, PSS-V1, AAD-by-PSS-V1interaction. Model 2: Alcohol measure AAD, tobacco use, marijuana use. Model 3: Alcohol measure AAD, AAD-by-PSS-V1 interaction, tobacco use, marijuana use, race, education, and Hispanic ethnicity.

## Data Availability

The data presented in this study are available on request from the corresponding author. The data are not publicly available due to lack of data sharing acknowledgement of de-identified data in the consent and IRB protocol.

## Supplementary Materials

The following supporting information can be downloaded at: https://www.mdpi.com/article/10.3390/ijms25052690/s1.
