# Supplementary material for: Independent and Combined Effects of Prenatal Alcohol Exposure and Prenatal Stress on Fetal HPA Axis Development
Source: Int J Mol Sci. 2024 Feb 26;25(5):2690. doi: 10.3390/ijms25052690 (PMC10932119; doi:10.3390/ijms25052690)
Supplement: Supplementary file 1 [file ijms-25-02690-s001.zip › ijms-2856010-supplementary.pdf]

International Journal of
Molecular Sciences

Table S1. Primer and Standard Oligo Sequences used in qPCR.

Gene	Primer Sequence	Source
**11β-HSD1**	FWD 5'- CTG AAG CAG AGC AAT GGA -3'<br>REV 5'- GCA GAA TAG GCA GCA ACC -3'	Validated by melt curve and agarose gel
Standard Sequence:	5'- CCA TGT GCG CAA AAG CAT GGA AGT CAA CTT CCT CAG TTA CGT GGT CCT GAC TGT AGC TGC CTT GCC CAT GCT GAA GCA GAG CAA TGG AAG CAT TGT TGT CGT CTC CTC TCT GGC TGG GAA AGT GGC TTA TCC AAT GGT TGC TGC CTA TTC TGC AAG CAA GTT TGC TTT GGA TGG GTT CTT CTC CTC CAT CAG AAA GGA ATA TTC AGT GTC CAG GGT CAA TGT A -3'	
**11β-HSD2**	FWD 5'- CTG TGA ACT CCT TCC CTG -3'<br>REV 5'- GAT GTA GTC CTT GCC GTA -3'	Validated by melt curve and agarose gel
Standard Sequence:	5'- TGC CAT ATC CGT GCT TGG GGG CCT ATG GAA CCT CCA AAG CGG CCG TGG CGC TAC TCA TGG ACA CAT TCA GCT GTG AAC TCC TTC CCT GGG GGG TCA AGG TCA GCA TCA TCC AGC CTG GCT GCT TCA AGA CAG AGT CAG TGA GAA ACG TGG GTC AGT GGG AAA AGC GCA AGC AAT TGC TGC TGG CCA ACC TGC CTC AAG AGC TGC TGC AGG CCT ACG GCA AGG ACT ACA TCG AGC ACT TGC ATG GGC AGT TCC TGC ACT CGC TAC GCC TGG CCA TGT CCG ACC TCA CCC CAG TTG TAG ATG -3'	
**CRH**	FWD 5'- GAA AGG AAG ACA ACC TCC AGA G -3'<br>REV 5'- ATG TTA GGG GCA CTC GCT T -3'	Validated by melt curve and agarose gel
Standard Sequence:	5'- AGA CCA AGT CCA TTG AGA GAC TGA GGG GAA AGA GAG GAG AGA AAG AAA AAG AGA GTG GGA ACA GTA AAG AGA AAG GAA GAC AAC CTC CAG AGA AAG CCC CCG GAG ACG TCT CTC TGC AGA GAG GCG GCA GCA CCC GGC TCA CCT GCG AAG CGC CTG GGA AGC GAG TGC CCC TAA CAT GCG GCT GCC GCT GCT TGT GTC CGC GGG AGT CCT GCT GGT GGC TCT CCT GCC CTG CCC GCC ATG CAG GGC G -3'	
**B2M**	FWD 5'- TGC TGT CTC CAT GTT TGA TGT ATC T -3'<br>REV 5'- TCT CTG CTC CCC ACC TCT AAG T -3'	[62]
Standard Sequence:	5'- AAT ATT GAT ATG CTT ATA CAC TTA CAC TTT ATG CAC AAA ATG TAG GGT TAT AAT AAT GTT AAC ATG GAC ATG ATC TTC TTT ATA ATT CTA CTT TGA GTG CTG TCT CCA TGT TTG ATG TAT CTG AGC AGG TTG CTC CAC AGG TAG CTC TAG GAG GGC TGG CAA CTT AGA GGT GGG GAG CAG AGA ATT CTC TTA TCC AAC ATC AAC ATC TTG GTC AGA TTT GAA CTC TTC AAT CTC TTG CAC TCA AAG CTT GTT AAG ATA GTT AAG CGT GCA TAA GTT AAC TTC CAA TTT ACA TAC TCT GCT TAG A -3'	
**GAPDH**	FWD 5'- TGC ACC ACC AAC TGC TTA GC -3'<br>REV 5'- GGC ATG GAC TGT GGT CAT GAG -3'	[62]
Standard Sequence:	5'- TCA TCA TCT CTG CCC CCT CTG CTG ATG CCC CCA TGT TCG TCA TGG GTG TGA ACC ATG AGA AGT ATG ACA ACA GCC TCA AGA TCA TCA GCA ATG CCT CCT GCA CCA CCA ACT GCT TAG CAC CCC TGG CCA AGG TCA TCC ATG ACA ACT TTG GTA TCG TGG AAG GAC TCA TGA CCA CAG TCC ATG CCA TCA CTG CCA CCC AGA AGA CTG TGG ATG GCC CCT CCG GGA AAC TGT GGC GTG ATG GCC GCG GGG CTC TCC AGA ACA TCA TCC CTG CCT CTA CTG GCG CTG CCA AGG CTG TGG GCA AGG T -3'	
**ARPC3**	FWD 5'- CCT GGT TTT CCA CTT AAC GCA -3'<br>REV 5'- AAT AGG CTC TCA TCA CTT CAT CTT -3'	[84]
Standard Sequence:	5'- ATT TCT GAA TGT CTG AAG AAA CTG CAA AAG TGC AAT TCC AAA AGC CAA GGT GAG AAA GAA ATG TAT ACG CTG GGA ATC ACT AAT TTT CCC ATT CCT GGA GAG CCT GGT TTT CCA CTT AAC GCA ATT TAT GCC AAA CCT GCA AAC AAA CAG GAA GAT GAA GTG ATG AGA GCC TAT TTA CAA CAG CTA AGG CAA GAG ACT GGA CTG AGA CTT TGT GAG AAA GTT TTC GAC CCT CAG AAT GAT AAA CCC AGC AAG TGG TGG ACT TGC TTT GTG AAG AGA CA -3'	

Table S2. Predictors of pCRH expression: sensitivity analysis.

	**PSS-V1**	**AAD**	**Tobacco**	**Marijuana**
	β (SE)	β (SE)	β (SE)	β (SE)
		**Gene pCRH (*N*=90)**		
Model 1	0.007(0.002)**	-0.06(0.15)	--	--
Model 2	0.007(0.002)**	0.02(0.16)	-0.05(0.08)	-0.05(0.04)
Model 3	0.007(0.002)**	0.002(0.16)	-0.04(0.08)	-0.04(0.04)

AAD, absolute alcohol (ounces) per day; PSS-V1, Perceived Stress Scale at visit 1.

** $p<0.01$.

Model 1: Alcohol measure AAD and PSS-V1.

Model 2: Alcohol measure AAD, PSS-V1, tobacco use, marijuana use.

Model 3: Alcohol measure AAD, PSS-V1, tobacco use, marijuana use, race, education, and Hispanic ethnicity.
